# Double Anti‐NMO and Anti‐MOG Positivity in a Patient With Metastatic Renal Carcinoma: First Reported Case

**DOI:** 10.1155/crnm/6191174

**Published:** 2026-01-26

**Authors:** M. Fortanet García, A. Belenguer Benavides, S. Blanco Madera, H. Benetó Andrés, A. Monclus Beclua, A. Recio Gimeno, L. Popova

**Affiliations:** ^1^ Department of Neurology, Castellon General University Hospital, Castellon de la Plana, Spain; ^2^ Pre-Departmental Unit of Medicine, Faculty of Health Sciences, Jaume I University, Castellon de la Plana, Spain, uji.es

**Keywords:** AQP4, corticosteroids, dual seropositivity, MOG, MOGAD, NMOSD, renal carcinoma, rituximab

## Abstract

**Background:**

Neuromyelitis optica spectrum disorder (NMOSD) and myelin oligodendrocyte glycoprotein‐associated disease (MOGAD) are central nervous system (CNS) demyelinating disorders characterized by autoantibodies targeting aquaporin‐4 (AQP4) and MOG, respectively. Although dual positivity for AQP4–IgG and MOG–IgG antibodies is uncommon, it poses significant diagnostic and therapeutic challenges due to its complex clinical features and uncertain prognosis.

**Case Presentation:**

We present the first reported case in the literature of a patient with metastatic renal carcinoma who tested positive for both AQP4 and MOG antibodies. A 49‐year‐old man with a history of metastatic renal carcinoma experienced progressive neurological symptoms, initially attributed to tumor progression. However, after further investigation, including lumbar puncture and autoantibody testing, a demyelinating process with dual seropositivity for AQP4–IgG and MOG–IgG was identified.

**Treatment and Outcomes:**

The patient was treated with high‐dose corticosteroids, followed by rituximab, resulting in clinical and radiological stability.

**Conclusion:**

This case highlights the rare occurrence of dual seropositivity for AQP4 and MOG antibodies in a patient with a history of metastatic renal carcinoma, which poses diagnostic and treatment challenges. Although the pathogenesis remains unclear, factors such as genetic predisposition and autoimmune coactivation may contribute to triggering this autoimmune response. This case emphasizes the importance of personalized treatment and the need for further research to optimize management strategies for patients with this complex condition.

## 1. Introduction

Autoantibodies targeting aquaporin‐4 (AQP4) [1] are considered the hallmark of neuromyelitis optica spectrum disorder (NMOSD), a collection of immune‐mediated disorders of the central nervous system (CNS) characterized by recurrent involvement of the optic nerve and/or spinal cord. The presence of AQP4 antibodies holds significant diagnostic and prognostic implications.

Alternatively, some patients with an NMOSD phenotype may harbor antibodies against myelin oligodendrocyte glycoprotein (MOG), which generally identifies a separate demyelinating disorder known as MOG antibody–associated disorders (MOGAD). NMOSD is typically classified as an immune‐mediated CNS disease. In ≥ 80% of cases, neuromyelitis optica (NMO) is caused by pathogenic IgG autoantibodies targeting AQP4. Approximately 10%–40% [1] of individuals with AQP4‐negative NMO have IgG autoantibodies directed against MOG.

Patients typically test positive for one of these antibodies, with double seropositivity being relatively rare [2]. Even when double seropositivity is observed, one of the two conditions may predominate, and the dominant phenotype can inform both treatment strategies and prognostic assessments. Clinical outcomes for NMO and MOG antibody–associated disease (MOGAD) are well documented. A diagnosis of NMO with AQP4 antibody positivity is associated with a significantly higher relapse rate [3] and a less favorable prognosis compared to AQP4‐negative NMO or isolated MOGAD.

However, the prognosis for double positivity remains unknown [4]. Future research on optic neuritis (ON) with double seropositivity is anticipated to offer valuable clinical insights and clarification regarding this distinctive condition.

We report a CNS demyelinating disease, exhibiting simultaneous positivity for both anti‐MOG and anti‐AQP4 antibodies in a patient diagnosed with metastatic renal carcinoma.

## 2. Case Description

We present a 49‐year‐old male patient with a history of metastatic renal carcinoma, diagnosed in December 2021, who is currently undergoing treatment with pembrolizumab and lenvatinib. A year and a half following diagnosis, the patient presented to the emergency department with a one‐week history of symptoms, including left‐sided hemibody and facial weakness. Various diagnostic tests were conducted: an electrocardiogram demonstrating sinus rhythm, normal blood gases, coagulation studies, complete blood count, a biochemistry panel, and a chest X‐ray within normal limits. A cranial computed tomography (CT) scan revealed a hypodense area in the right corona radiata suggestive of metastasis. Given the suspicion of tumor progression, the patient was transferred to an oncology center for further studies.

At this healthcare facility, additional diagnostic evaluations comprised a routine blood count and biochemistry analysis. A CT scan of the chest, abdomen, and pelvis revealed a reduction in the size of a supraclavicular lymph node. There was stability observed in the bone metastases and the renal mass. Additionally, mild dilation of the small intestine was noted.

A supra‐aortic trunk ultrasound and transthoracic echocardiography yielded normal results. The bone scan demonstrated no significant abnormalities. Brain magnetic resonance imaging (MRI) revealed a subacute infarction in the right choroidal and lenticulostriate arteries.

**Figure 1:**
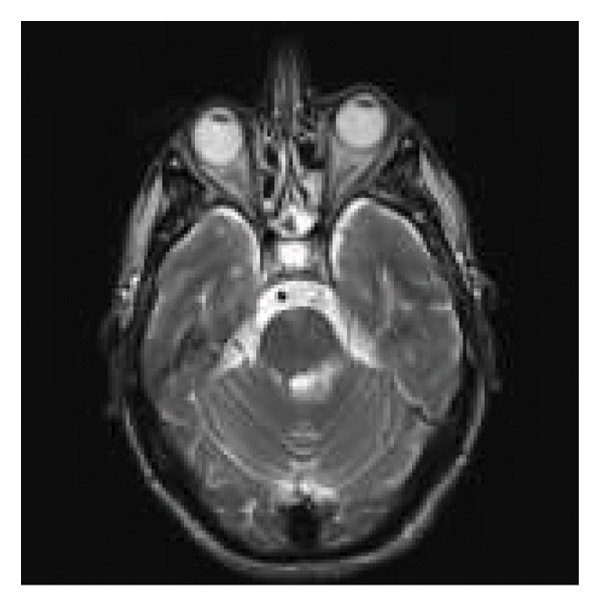
Axial brain MRI, FLAIR sequence, showing several hyperintense lesions, the largest located at the left cerebellar peduncle.

A month later, the patient was admitted to our neurology department due to progressive neurological worsening. Over the previous month, he had developed gait instability, liquid dysphagia, hiccups, worsening speech, and diplopia. Neurological examination showed mild dysarthria, left eye esotropia, vertical nystagmus, moderate left lower facial paresis, and severe left hemiparesis. Brain MRI revealed two areas of vasogenic edema with contrast enhancement: one on the slope of the left middle cerebellar peduncle and another in the right corona radiata, the caudate body/head, and the upper half of the ipsilateral lenticular nucleus, suggesting intraparenchymal and leptomeningeal tumor dissemination (Figure 1). During admission, dexamethasone was administered, resulting in clinical improvement. The case was discussed in the neuro‐oncology committee, where it was decided to transfer the patient back to the oncology hospital due to suspected tumor involvement to continue treatment and receive care from his primary oncology team.

During this second admission, it was considered that the lesions might have an inflammatory rather than a tumoral origin. Based on this suspicion, a lumbar puncture was performed, which showed negative results for tumor cells, and an autoimmunity panel was ordered, revealing positive anti‐MOG antibodies with a titer of 1:160 (detected via indirect immunofluorescence) and positive anti‐NMO IgG at 0.15 (detected via enzyme‐linked immunoassay), supporting the diagnostic hypothesis. Treatment was initiated with methylprednisolone, followed by maintenance with prednisone.

In a subsequent antibody evaluation performed at our facility two months later, anti‐AQP4 antibodies tested negative, whereas anti‐MOG antibodies tested positive with a titer of 1:10 (both antibodies identified via indirect immunofluorescence).

**Figure 2:**
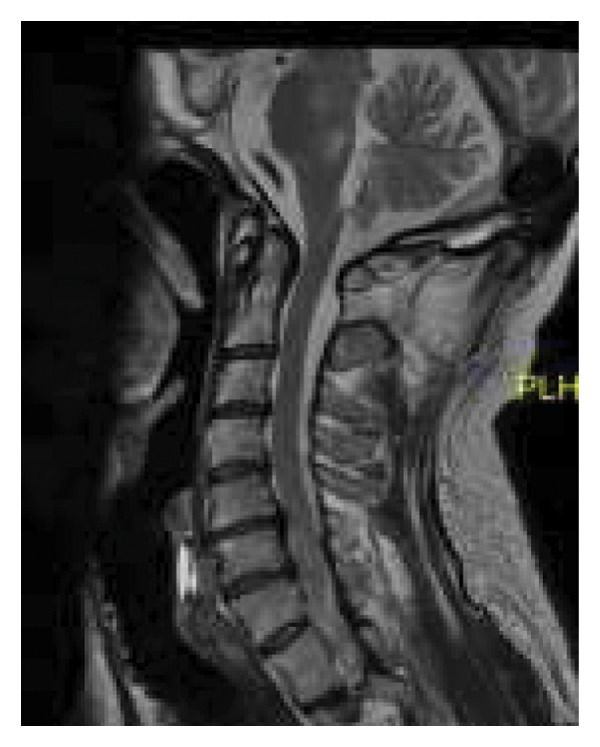
Cervical spinal MRI, T2 sagittal view. Hyperintense image at C5–C6.

In September 2023, the patient was readmitted due to clinical deterioration following the cessation of corticosteroid therapy. High‐dose methylprednisolone was administered, resulting in improvement. Brain MRI performed at this time demonstrated a reduction in edema and contrast enhancement within the lesions, along with the emergence of new demyelinating lesions in the cervical spinal cord at the C5–C6 level (Figure 2).

**Figure 3:**
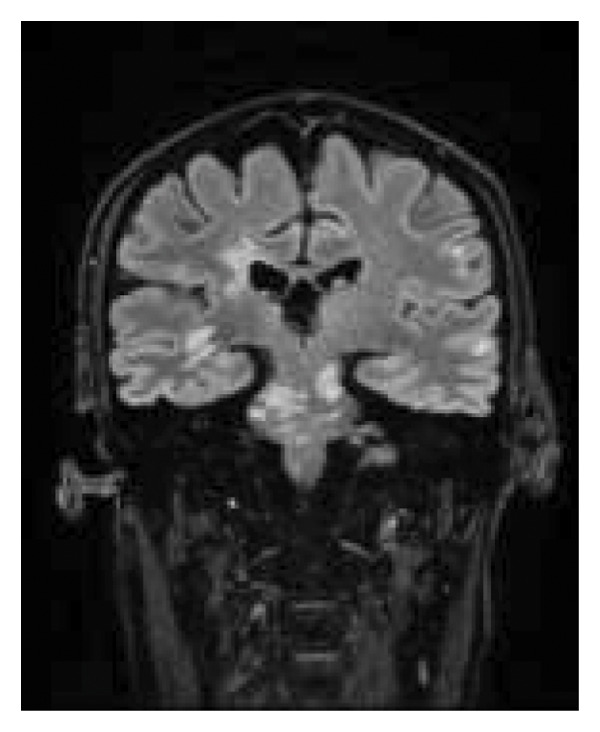
Coronal brain MRI, FLAIR sequence. Radiological worsening following corticosteroid tapering.

A month later, rituximab was initiated following antituberculosis prophylaxis due to a positive IGRA test. However, after the second induction dose and a reduction in prednisone dosage, the patient experienced neurological worsening. MRI revealed new bilateral subcortical inflammatory lesions in the left midbrain and the right cerebellar peduncle (Figure 3). High‐dose corticosteroids were reintroduced, resulting in clinical improvement. A follow‐up MRI demonstrated stability in the demyelinating lesions. At the time of discharge, the patient was in a good general condition: eupneic, alert, oriented in all spheres, attentive, and cooperative. Speech was free of dysphasic features, with mild dysarthria. Extraocular movements were preserved, with no nystagmus or diplopia. Mild left facial asymmetry was observed. There was mild spastic left hemiparesis, predominantly affecting the arm (4/5 strength), with 5/5 strength in the right limbs. The left plantar reflex was extensor. Mild dysmetria was noted in the left limbs, along with paresthesia in the hands, without hypoesthesia or sensory extinction. No clonus was observed. Reflexes were hyperactive in the left hemibody.

**Figure 4:**
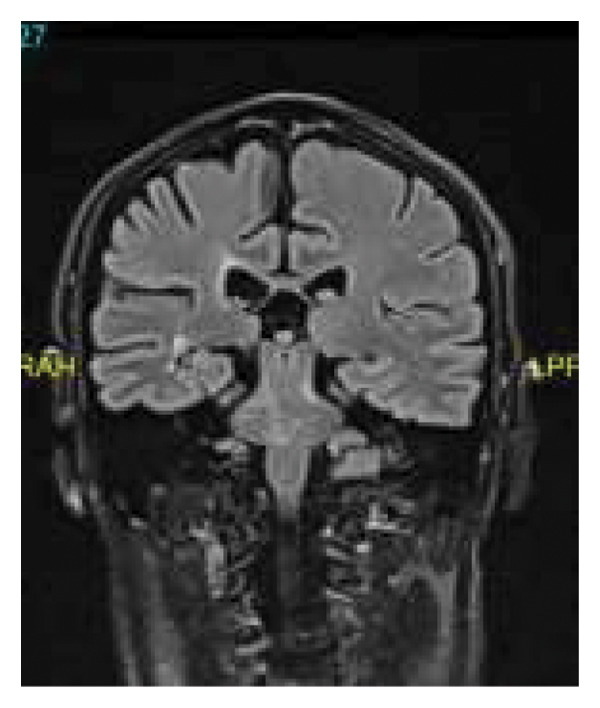
Coronal brain MRI, FLAIR sequence. Posttreatment radiological improvement.

In February 2024, during an outpatient consultation, corticosteroid therapy was discontinued, and the patient did not require additional hospitalization. His clinical and radiological findings have demonstrated stabilization under immunosuppressive therapy, indicating a primary demyelinating disorder with pseudotumoral features and dual positivity for anti‐MOG and anti‐AQP4 antibodies (Figure 4).

## 3. Discussion

AQP4 IgG–associated NMOSD (AQP4–IgG NMOSD) and MOGAD are both distinctive and clinically separate inflammatory demyelinating conditions of the CNS, characterized by specific clinical, serologic, and radiologic features [1].

AQP4–IgG NMOSD is widely regarded as an immune‐mediated disorder of the CNS, distinguished by the recurrent involvement of the optic nerve and/or spinal cord.

In more than 80% of cases [1], NMOSD is attributed to pathogenic IgG autoantibodies targeting AQP4, which hold diagnostic and prognostic significance. Additionally, approximately 10%–40% of patients with AQP4‐negative NMOSD demonstrate the presence of MOG‐directed IgG autoantibodies.

The incidence and prevalence of NMOSD differ across various racial and ethnic populations. The lowest prevalence is observed among Caucasians at a rate of 1 per 100,000 individuals, whereas the highest prevalence occurs among Black populations at 10 per 100,000 individuals [5]. A study conducted in Mangalore, South Asia, indicated that 13.9% of all demyelinating disorder cases were NMOSD, with a prevalence of 2.6 per 100,000 persons [6].

Few cases of nonparaneoplastic NMOSD with dual seropositivity of AQP4 and MOG antibodies have been described. In one study [7], 10 of 125 patients with NMOSD (8%) were double positive for AQP4–IgG and MOG–IgG. These double‐positive patients had a multiphasic disease course with a high annual relapse rate and severe residual disability. A study of 1935 cases of NMOSD [4] found double positivity (AQP4 antibody + MOG antibody) in 3 cases (0.15%). Their clinical phenotype was more similar to NMOSD than to MOGAD.

Although NMOSD is primarily classified as an idiopathic autoimmune disease, it can also manifest as a paraneoplastic syndrome in approximately 1.3%–25% of patients [8]. Patients with paraneoplastic NMOSD are generally older at the time of disease onset, with a mean age ranging from 50 to 55 years, compared to the overall mean age of onset for NMOSD, which ranges from 32 to 41 years. Paraneoplastic NMOSD predominantly affects women (70.6%–91%) [8], although this proportion is comparatively lower than that observed in idiopathic NMOSD.

It is hypothesized that tumor onconeural antigens stimulate the host immune system to produce antibodies such as AQP‐4 IgG, which crossreact with nervous tissue and induce CNS damage. Breast and lung cancers are the two most frequent tumors that trigger paraneoplastic NMOSD, with breast cancer accounting for 18.3%–32% of all cases.

Other neoplasms documented in the literature include lymphoma, gastric carcinoid, esophageal carcinoma, teratoma, multiple myeloma, and ovarian carcinoma. The demonstration of AQP‐4 or MOG expression within the tumor via immunohistochemical staining is essential to establish a causal association [9]. A limitation of our case study is the absence of assessment of antibody expression by the tumor cells, which prevents us from confirming a causal relationship.

The prevalence of paraneoplastic NMOSD among cancer patients with positive AQP4–IgG antibodies is approximately 0.02% [10].

Sepulveda [11] and other authors have documented that the most common presenting symptoms in patients with paraneoplastic NMOSD were severe nausea and vomiting, which were observed to be significantly more prevalent in these patients compared to those without malignancies (41.2% vs. 6.6%). Furthermore, they identified several risk factors for paraneoplastic NMOSD, including presentation with postrema involvement (intractable nausea and vomiting), age exceeding 45 years, and male gender.

Patients typically test positive for one of these antibodies, with dual seropositivity being uncommon. Even in cases of dual seropositivity, one pathology may predominate, and the dominant phenotype can inform both treatment decisions and prognosis [12]. In our case, despite the presence of dual seropositivity, several features suggest a phenotype more aligned with MOGAD: persistent MOG positivity, tumefactive brain lesions with brainstem involvement, a favorable response to corticosteroids, and relapse following tapering—all characteristic of MOGAD [13]. Conversely, classical NMOSD typically manifests with ON, longitudinally extensive transverse myelitis (LETM), and area postrema syndrome [14]. The absence of data regarding dual seropositive cases has resulted in a noticeable gap in comprehending their pathophysiology, presenting characteristics, and prognosis.

The incidence of dual seropositivity has demonstrated variability, ranging from 0% to 26%, in previous studies, contingent upon the testing methodology employed [2]. In a particular study [14], Kezuka et al. observed that 6 out of 23 patients (26%) exhibited seropositivity for AQP4 antibodies and MOG antibodies. Conversely, Höftberger R et al. reported that, among 174 patients, only two (1.1%) were dually seropositive [15]. However, a study conducted by Höftberger et al. [16], involving 215 patients, identified no cases of dual seropositivity. Additional research [12–17] also failed to detect instances of concurrent antibody seropositivity.

In our case, AQP‐IgG antibodies were initially positive; however, subsequent testing conducted at our center yielded negative results. This discrepancy may be attributed to the differing methodologies employed for antibody detection at the respective centers where the analyses were performed. While ELISA is commonly utilized in clinical practice for the detection of AQP4–IgG antibodies and demonstrates acceptable sensitivity, cell‐based assays (CBA) exhibit superior sensitivity and specificity for these antibodies. Consequently, the inconsistency between the results of the two assays raises the likelihood of an initial false‐positive ELISA outcome or technical interference related to the assay, thereby creating uncertainty regarding the patient’s true AQP4–IgG serostatus. Therefore, discrepancies between assay results, as observed in our patient, may reflect methodological variability or limited reproducibility across different platforms. However, a secondary origin of AQP4–IgG may stem from extensive astrocytic damage caused by myelitis of alternative etiology. In such cases, the presence of AQP4–IgG would be transient [18]. This underscores the diagnostic challenges posed by these pathologies and emphasizes the importance of close follow‐up and continuous monitoring, taking into account methodological factors, assay sensitivity, and reproducibility within each individual case.

With regard to MOG–IgG antibody titers, our patient exhibited a decline from 1:160 to 1:10. This observation holds clinical significance, as decreasing titers and eventual seronegatization have been correlated with a reduced risk of relapse, whereas persistently high titers are associated with a relapsing disease course [19]. However, it is important to note that low titers are less specific, may fluctuate with treatment, and display variability across different assays. Although the downward trend supports the impression of disease stabilization, ongoing clinical, radiological, and serological monitoring remains imperative, as relapses may still occur despite low or declining titers [20].

A diagnosis of NMO with AQP4–IgG positivity indicates a significantly increased relapse rate and an unfavorable prognosis in comparison to NMO cases testing negative for AQP4–IgG or those with isolated MOGAD. Nevertheless, the prognosis for cases exhibiting double positivity remains uncertain.

These two antibodies have distinct immunopathogenesis, with variable clinical phenotypes and prognoses. Their synergistic effect needs to be better documented.

The pathophysiology underlying the coexistence of these antibodies remains unclear. CNS cells that express MOG are distinct from those expressing AQP4. Nonetheless, microglial cells, functioning as antigen‐presenting cells, possess the capacity to “cross‐present” both MOG and AQP4 [14].

It has also been postulated that co‐occurrence is an epiphenomenon; however, if this was the case, the antibodies would be found to coexist more frequently. Therefore, the rarity itself is evidence against an epiphenomenon and suggests immunopathogenic differences between these disorders [20].

Another potential explanation for the coexistence is the inherent tendency of autoimmune disorders to promote the development of additional autoantibodies, along with a genetic predisposition to produce autoantibodies targeting multiple organs or tissues [4].

Furthermore, cases of NMOSD have been associated with immunotherapy treatments, including pembrolizumab [21], nivolumab [22], or a combination of multiple immune checkpoint inhibitors (ICIs) [23]. The immune response provoked by an ICI can be misdirected, leading to an autoimmune phenomenon that may occasionally affect the nervous system [10]. Although the exact pathophysiological mechanism remains inadequately understood, several hypotheses have been proposed. One hypothesis suggests that ICI‐induced activation of Type 2 helper T cells results in B‐cell stimulation, subsequently producing AQP4–IgG [22]. Alternatively, ICIs may trigger an autoimmune response that cross‐reacts with antigens in the CNS, thereby supporting a paraneoplastic process. Another possibility is that ICIs independently stimulate immune responses targeting both the tumor and the CNS. Finally, ICIs might directly recognize their respective receptors (CTLA4, PD‐1 [programmed death‐1], or PD‐L1) on CNS cells such as neurons, astrocytes, and endothelial cells, thus directly inducing a complement‐mediated or cell‐mediated cytotoxic inflammatory response [23]. Correspondingly, neurological immune‐related adverse events (n‐irAEs) are generally uncommon; systematic reviews and meta‐analyses estimate an incidence of approximately 1%–6% for anti‐PD‐1/PD‐L1 agents, with CNS events being less frequent than neuromuscular syndromes. Further research is necessary to elucidate the underlying pathophysiological mechanisms.

ICI‐associated NMOSD typically occurs from two weeks to 11 months after ICI initiation, with the risk increasing in cases involving combined ICI regimens [24]. Consistently, multiple cohorts report that most neurological toxicities manifest early, usually within 1–4 months (often around 6–8 weeks) from the first ICI dose; median onset is frequently cited around 4–9 weeks, and encephalitis peaks near two months [23, 25]. Combination ICI therapy (e.g., anti‐CTLA‐4 plus anti‐PD‐1) confers a higher risk of neurological toxicity compared to monotherapy. Pembrolizumab targets PD‐1, with neurological adverse events related to anti‐PD‐1/PD‐L1 occurring in 6.1% of cases, and the median onset of neurotoxicity is three months [10]. Although isolated cases of AQP4–IgG NMOSD have been reported shortly after pembrolizumab or other ICIs (often within months of initiation), these instances remain exceptional [26]. In the present case, the cause is considered unlikely to be related to immunotherapy because pembrolizumab was commenced shortly after the diagnosis of renal cancer, and the neurological symptoms did not appear until two years later. This prolonged latency markedly exceeds the commonly reported timeframes for n‐irAEs; consensus guidelines note that new neurologic autoimmunity occurring more than 12 months after the last ICI exposure is generally not consistent with an irAE. Recent series on late‐onset irAEs demonstrates that very delayed presentations are uncommon and more often involve nonneurological manifestations [25].

In the study by Spiezia AL et al., a total of 30 cases exhibiting both anti‐MOG and anti‐AQP4 positivities were documented in the literature up to the year 2023 (see Supporting Table S1) [1]. The majority of these patients were female (12 out of 13), presented with high‐grade disability (EDSS > 6, 9 out of 13), and demonstrated extensive involvement of the spinal cord. Specifically, six patients were diagnosed with LETM, two with ON and five with both LETM and ON. The neuroimaging findings revealed that most patients (11 out of 13) had lesions in the brain, whereas only two exhibited normal neuroimaging results. These findings are consistent with a study conducted by Yan Y et al., where women constituted a larger proportion of dual‐positive patients, and recurrent disease was observed in 100% of cases with dual seropositivity. [7].

In accordance with cases documented in the literature, our patient demonstrated involvement of the cerebral and cervical spinal cord. However, contrary to most reported cases, he did not exhibit optic nerve involvement, as observed in the case by Miyagishima D et al. [8].

Finally, immunotherapy‐based regimens are employed for the treatment of these cases. During the acute phase, high‐dose methylprednisolone, immunoglobulins, or plasmapheresis are typically administered. For maintenance therapy, the most frequently utilized agents are rituximab or azathioprine; however, there have been reported instances involving hydroxychloroquine [27], subcutaneous ofatumumab [28], or eculizumab [29]. In our case, methylprednisolone was administered during the acute phase, with prednisone serving as a transitional therapy to initiate rituximab as maintenance immunosuppressive treatment, in accordance with established guidelines from relevant case reports in the literature.

## 4. Conclusions

The concurrent positivity for AQP4–IgG and MOG–IgG antibodies in CNS demyelinating disorders is an uncommon occurrence. It poses significant diagnostic and therapeutic challenges owing to its multiphasic progression and elevated relapse rates. Although data regarding its pathophysiology are limited, it is hypothesized that genetic predispositions and external factors, such as ICIs, may induce a cross‐reactive autoimmune response, thereby activating both antibodies.

This condition appears to amalgamate features of NMOSD and MOGAD; however, individual cases may exhibit a predominant phenotype that informs treatment strategies. The concurrent presence of these antibodies, particularly in the context of tumors or oncological treatments, highlights the necessity of a thorough and individualized evaluation, especially for patients with a history of oncological illness.

The management of these cases primarily relies on immunotherapy, including corticosteroids and rituximab. However, variability in treatment response underscores the necessity for close monitoring and further research to more comprehensively understand the underlying immunological mechanisms, with the ultimate goal of optimizing therapeutic approaches for patients presenting this complex clinical picture.

## Ethics Statement

Ethical review and approval were waived for this study, as it is a case report and a review of the available literature, without the inclusion of any personal data that could identify the patient.

## Disclosure

All authors have read and agreed to the published version of the manuscript.

## Conflicts of Interest

The authors declare no conflicts of interest.

## Author Contributions

Conceptualization: M. Fortanet García and A. Belenguer Benavides; methodology: A. Belenguer Benavides and M. Fortanet García; data collection: M. Fortanet García, A. Belenguer Benavides, H. Benetó Andrés, A. Recio Gimeno, L. Popova, and S. Blanco Madera; resources: M. Fortanet García and A. Belenguer Benavides; data curation: S. Blanco Madera, H. Benetó Andrés, A.M.M, A. Recio Gimeno, and L. Popova; writing–original draft preparation: M. Fortanet García and A. Belenguer Benavides; writing–review and editing: M. Fortanet García and A. Belenguer Benavides.

## Funding

This research received no external funding.

## Supporting Information

Supporting Table S1 provides a comprehensive overview of the clinical, radiological, and therapeutic features of previously documented patients exhibiting dual positivity for aquaporin‐4 (AQP4) and myelin oligodendrocyte glycoprotein (MOG) antibodies. The table encompasses demographic information, initial clinical presentation, relapse history, disability assessments, associated autoantibodies, MRI findings of the brain and spinal cord, and the treatments administered. These data have been derived from published case reports and case series available in the literature up to 2023 and are intended to offer contextual support for the findings discussed in the present study.

## Supporting information


**Supporting Information** Additional supporting information can be found online in the Supporting Information section.

## Data Availability

For this publication, data obtained from the existing literature of similar reported cases have been used, which are available at the locations cited in the “References” section of this document.
