# Supplementary material for: Double Anti‐NMO and Anti‐MOG Positivity in a Patient With Metastatic Renal Carcinoma: First Reported Case
Source: Case Rep Neurol Med. 2026 Jan 26;2026:6191174. doi: 10.1155/crnm/6191174 (PMC12835620; doi:10.1155/crnm/6191174)
Supplement: Supplementary file 1 — Supporting Information Additional supporting information can be found online in the Supporting Information section. [file CRNM-2026-6191174-s001.docx]

| Study | Sex | Age | Onset Phenotype | N° Relapses | Current EDSS | Other Antibody | Spine Lesion | Brain Lesion | Treatment |
| --- | --- | --- | --- | --- | --- | --- | --- | --- | --- |
| Hyun et al., 2017 | M | 32 | LETM | 4 | 7 | Anti-SS-A Ab (+), antinuclear Ab (+), speckled pattern | NA | None | AZA |
| Höffberger et al., 2014 | F | 58 | LETM + ON | 1 | 2 | No | C1-C5 | None | RTX |
|  | F | 50 | LETM + ON | 1 | 2 | No | C4-T2 | Aspecific WML | AZA |
|  | F | 15 | LETM + ON | 3 | 7 | No | C4-conus | Large MS-like lesions | CS, AZA |
|  | F | 36 | LETM | 8 | 8 | No | T1-conus | Small MS-like lesions | RTX |
| Yan et al., 2016 | F | 18 | LETM + ON | 8 | 8.5 | Anti-SS-A/AB, Anti-Tg, anti-TPO | C1-conus | Small MS-like lesions | Met HCQ |
|  | F | 60 | LETM | 6 | 8.5 | No | C2-conus | Small MS-like lesions | CS, AZA |
|  | F | 49 | LETM | 3 | 7.5 | No | C1-conus | Small MS-like lesions | CS, AZA |
|  | F | 33 | ON | 6 | 3 | No | C3-conus | Large MS-like lesions | CS, AZA |
|  | F | 15 | ON | 7 | 3 | No | C1-conus | ADEM-like | CS, AZA |
|  | F | 20 | LETM + ON | 10 | 6.5 | No | C1-conus | ADEM-like | CS, AZA |
|  | F | 30 | LETM | 4 | 7.5 | No | C1-conus | ADEM-like | RTX |
|  | F | 36 | LETM | 6 | 8 | No | C1-T12 | Small MS-like lesions | RTX |
| Ishikawa et al., 2019 | F | 24 | ON | 3 | NA | NA | NA | NA | CS, IV Ig, AZA |

Ab, antibody; ADEM, acute disseminated encephalomyelitis; AZA, azathioprine; CS, corticosteroids; EDSS, Expanded Disability Status Scale; F, female; HCQ, hydroxychloroquine; IV Ig, intravenous immunoglobulins; JC, juxtacortical white matter; LETM, longitudinally extensive transverse myelitis; M, male; Met, methotrexate; MS, multiple sclerosis; NA, not available; NMOSD, neuromyelitis optica spectrum disorder; ON, optic neuritis; RTX, rituximab; Tg, thyroglobulin; TPO, thyroperoxidase; WML, white matter lesion.

Supplementary table S1, derived from the article by Spieza AL et al., summarizes the clinical and radiological characteristics of AQP4 and MOG dual-positive cases reported in the literature up to 2023.
